# *Schistosoma mansoni* glyceraldehyde-3-phosphate dehydrogenase enhances formation of the blood-clot lysis protein plasmin

**DOI:** 10.1242/bio.050385

**Published:** 2020-03-24

**Authors:** David B. Pirovich, Akram A. Da'dara, Patrick J. Skelly

**Affiliations:** Molecular Helminthology Laboratory, Department of Infectious Disease and Global Health, Cummings School of Veterinary Medicine, Tufts University, North Grafton, MA 01536, USA

**Keywords:** Schistosome, Tegument, GAPDH, Moonlighting function, Thrombolysis

## Abstract

Schistosomes are intravascular blood flukes that cause the parasitic disease schistosomiasis. In agreement with *Schistosoma mansoni* (Sm) proteomic analysis, we show here that the normally intracellular glycolytic enzyme glyceraldehyde-3-phosphate dehydrogenase (GAPDH) is also found at the parasite surface; live worms from all intravascular life stages display GAPDH activity. Suppressing GAPDH gene expression using RNA interference significantly lowers this live worm surface activity. Medium in which the worms are cultured overnight displays essentially no activity, showing that the enzyme is not shed or excreted but remains associated with the worm surface. Immunolocalization experiments confirm that the enzyme is highly expressed in the parasite tegument (skin). Surface activity in schistosomula amounts to ∼8% of that displayed by equivalent parasite lysates. To address the functional role of SmGAPDH, we purified the protein following its expression in *Escherichia* *coli* strain DS113. The recombinant protein displays optimal enzymatic activity at pH 9.2, shows robust activity at the temperature of the parasite's hosts, and has a Michaelis–Menten constant for glyceraldehyde-3-phosphate (GAP) of 1.4 mM±0.24. We show that recombinant SmGAPDH binds plasminogen (PLMG) and promotes PLMG conversion to its active form (plasmin) in a dose response in the presence of tissue plasminogen activator. Since plasmin is a key mediator of thrombolysis, our results support the hypothesis that SmGAPDH, a host-interactive tegumental protein that can enhance PLMG activation, could help degrade blood clots around the worms in the vascular microenvironment and thus promote parasite survival *in vivo*.

This article has an associated First Person interview with the first author of the paper.

## Abstract (precis)

**Summary:** This work provides evidence of a novel function for a blood fluke's glycolytic enzyme (GAPDH) activating plasmin to potentially degrade blood clots, thus aiding parasite survival in the host vasculature.

## INTRODUCTION

Schistosomiasis is a debilitating parasitic infection caused by trematode worms of the genus *Schistosoma.* Infection in humans is primarily attributed to three *Schistosoma* species: *Schistosoma* *mansoni*, *Schistosoma haematobium* and *Schistosoma* *japonicum* (Colley et al., 2014; McManus et al., 2018). Over 200 million people worldwide – with a majority residing in Africa – are afflicted with schistosomiasis, and nearly 800 million more are at risk of infection (Lewis and Tucker, 2014; McManus et al., 2018; Vale et al., 2017). Behind malaria, schistosomiasis is considered the second most socioeconomically burdensome parasitic disease on the planet, and kills over 250,000 individuals annually in sub-Saharan Africa alone (Lewis and Tucker, 2014; Nour, 2010; van der Werf et al., 2003). Infection occurs when larval parasites (cercariae) emerge from freshwater snail intermediate hosts and penetrate the skin of the definitive human host. Inside the body, the parasites transform into juveniles called schistosomula. These larvae invade the vasculature where they mature into adults and mate. Adults can live in the host bloodstream for many years and, despite being obstacles to blood flow, appear not to elicit damaging blood clot formation around them (Gryseels et al., 2006; Keating et al., 2006; Wang et al., 2017). Several mechanisms have been proposed by which schistosomes might inhibit blood clotting (Elzoheiry et al., 2019, 2018b,a; Mebius et al., 2013; Wang et al., 2018, 2017). For instance, the worms possess a series of ectoenzymes that are thought to impact this process: the surface diphosphohydrolase SmATPDase1 and the surface phosphodiesterase/pyrophosphatase SmNPP5 can both cleave the platelet activator adenosine diphosphate (ADP) (Elzoheiry et al., 2018b), and, as shown for SmNPP5, this can block platelet aggregation *in vitro* (Elzoheiry et al., 2018b). The surface ectoenzyme alkaline phosphatase SmAP can cleave the pro-coagulant lipid mediator sphingosine-1-phosphate (Elzoheiry et al., 2018a) as well as the prothrombotic polymer polyphosphate (polyP) (Elzoheiry et al., 2019). In addition, host-interactive tegumental proteases can cleave key components of the coagulation cascade such as fibronectin (Wang et al., 2017) and high-molecular-weight kininogen (Wang et al., 2018).

It has additionally been proposed that schistosomes can hijack components of the host's own system of blood clot dissolution to aid thrombolysis (Mebius et al., 2013). Under normal conditions, thrombolysis begins when the zymogen plasminogen (PLMG) is converted by e.g. tissue plasminogen activator (tPA) into its enzymatically active form, plasmin – a serine protease that hydrolyses cross-linked fibrin (a major molecular component of blood clots) (Figuera et al., 2013). We previously showed that live intravascular-stage schistosome parasites (schistosomula and adult males and females) can all promote significant PLMG activation in the presence of tPA, which results in rapid plasmin generation (Figueiredo et al., 2015). In addition, it was demonstrated that the *S. mansoni* glycolytic enzyme enolase (SmEno), in addition to being widely distributed in the internal tissues of schistosomes, also exists in a host-interactive tegumental form (Figueiredo et al., 2015). Further, recombinant SmEno (rSmEno) was shown to bind PLMG and promote its conversion to plasmin, in the presence of tPA (Figueiredo et al., 2015). Suppressing expression of the SmEno gene significantly diminished enolase mRNA levels, protein levels and surface enolase activity but, somewhat surprisingly, did not appreciably affect the ability of live worms to promote PLMG activation (Figueiredo et al., 2015). Thus, while SmEno could enhance PLMG activation, our analysis showed that it was not the only contributor to the parasite's ability to perform this function (Figueiredo et al., 2015). Indeed, in the ruminant parasite *Schistosoma bovis* several proteins that are found in tegumental extracts (and including enolase) all bind PLMG, showing that there can be great redundancy regarding this molecular function (Ramajo-Hernández et al., 2007). One such *S. bovis* protein is the glycolytic enzyme glyceraldehyde-3-phosphate dehydrogenase (GAPDH) (Ramajo-Hernández et al., 2007). GAPDH is best known as a key enzyme in glycolysis, facilitating the conversion of glyceraldehyde-3-phosphate (GAP) in the presence of nicotinamide adenide dinucleotide (NAD) to 1,3-bisphosphoglycerate (1,3BPG) and generating NADH (Seidler, 2013). *S. mansoni* GAPDH (SmGAPDH) has been previously expressed in an enzymatically active recombinant form (Argiro et al., 2000a; El Ridi et al., 2001, 2004), has been characterized as a potential vaccine candidate and has been shown to be a target for antibodies in the sera of schistosomiasis-resistant individuals (Argiro et al., 2000a,b; Dessein et al., 1988; Goudot-Crozel et al., 1989; Tallima et al., 2017; Tang et al., 2019).

While SmGAPDH is found within all schistosome tissues, the protein has also been identified in several tegumental proteomic studies (Braschi et al., 2006; Braschi and Wilson, 2006; Sotillo et al., 2015; van Balkom et al., 2005), suggesting that it is additionally located on the parasite's external surface. Indeed, previous immunofluorescence studies using live parasites have localized GAPDH to the apical surface of the parasite (Goudot-Crozel et al., 1989; Tallima and El Ridi, 2008). We set out here to determine if functional GAPDH was detectable at the host-parasite interface. Our hypothesis is that, at the parasite surface, the enzyme might engage in non-glycolytic ‘moonlighting’ functions such as PLMG activation, which could promote worm survival.

## RESULTS

### Live schistosomes exhibit functional surface GAPDH activity

**Fig. 1.**
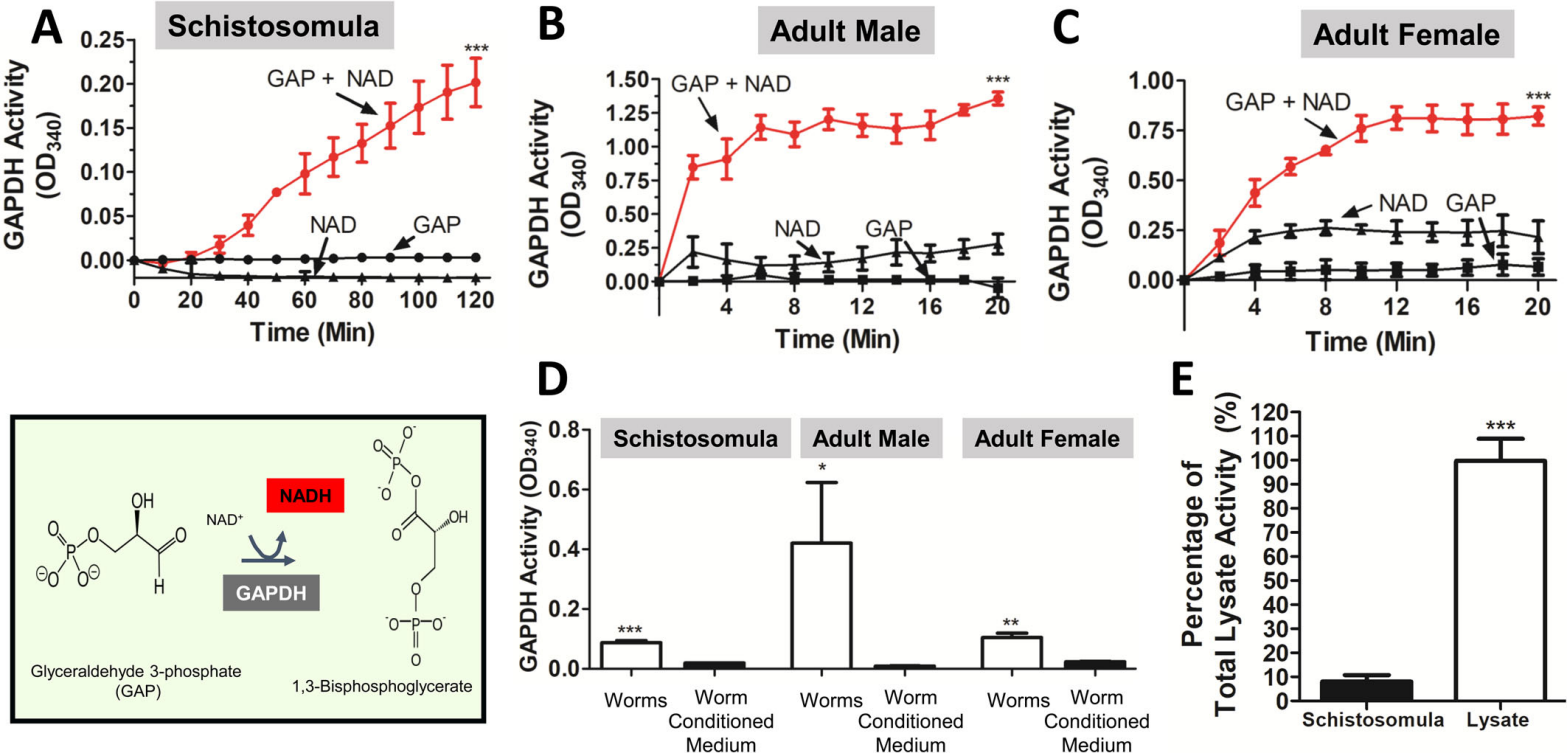
**Characterization of native SmGAPDH.** The reaction catalyzed by GAPDH (the sixth step of glycolysis) – glyceraldehyde 3-phosphate (GAP) conversion to 1,3-bisphosphogylcerate and the reduction of NAD to NADH – is illustrated in the box on the lower left. (A–C) NADH generation over time (mean of triplicate OD_340_ values±s.e.m.) by live schistosomula (in groups of 500) (A) or adult male pairs (B) or adult female pairs (C) when incubated with GAP and NAD is depicted (red lines). NADH generation following worm incubation with only GAP or NAD is also depicted (black lines). (D) NADH generation (mean of triplicate OD_340_ values±s.e.m. at 60 min) by live schistosomula (in groups of 500), adult male pairs and adult female pairs following their overnight incubation in clear medium (and in the presence of GAP and NAD) compared with NADH generation in the conditioned overnight medium itself. (E) NADH generation (mean of triplicate OD_340_ values±s.e.m. at 10 min) of live schistosomula (in groups of 500) compared to NADH generation by an equivalent schistosomula lysate. Lysate values are set at 100%. All assays were replicated at least three times. Significant differences in NADH detection at endpoint are denoted by ***P<0.001, **P<0.01, *P<0.05 [two-way ANOVA with Bonferroni post-tests (A–C) or one-tailed unpaired *t*-test (D)].

In the sixth step of the ten-step glycolytic pathway, GAPDH catalyzes the conversion of its substrate glyceraldehyde 3-phosphate (GAP) to 1,3-bisphosphogylcerate.(Seidler, 2013) The reaction, depicted in Fig. 1, lower left box, also leads to the conversion of NAD to its reduced form NADH and this molecule can be detected at optical density at 340 nm wavelength (OD340) (Argiro et al., 2000a,b; Ferdinand, 1964; Seidler, 2013). To monitor surface GAPDH activity in live *S. mansoni* schistosomula and adult parasites, intact, living worms are incubated in the presence of GAP and NAD in assay buffer (pH 9.2), and any NADH generated is detected spectrophotometrically. Changes in OD_340_ over time represent NADH generation by functional enzyme. As assessed by this assay, Fig. 1 shows that all three life stages – schistosomula (Fig. 1A), adult males (Fig. 1B) and adult females (Fig. 1C) – exhibit robust GAPDH activity. Significant differences in NADH detection were noted at the endpoint of each activity assay (*P*<0.001).

To determine if the GAPDH activity seen in Fig. 1A–C was because the enzyme had been secreted by the worms (or had been released because of damage to the worms in culture), GAPDH activity was tested in conditioned culture medium in which parasites had been incubated overnight. After overnight incubation, the parasites were removed, an equal volume of GAP and NAD in assay buffer (pH 9.2) was added and NADH generation was monitored at OD_340_. Recovered parasites were assayed in fresh medium [to which GAP and NAD in assay buffer (pH 9.2) was added] for parallel enzyme activity testing. As earlier, live worms exhibited robust GAPDH activity whereas just trace changes in OD_340_ were detected in the overnight medium. This was the case for schistosomula as well as adult males and adult females (Fig. 1D). The surface GAPDH activity of live schistosomula was next compared to the total GAPDH activity displayed by whole-parasite lysates. To do this, the activity detected in groups of 1000 live schistosomula was compared to the activity detected in homogenates of an equivalent number of schistosomula and results are shown in Fig. 1E. Mean surface GAPDH activity was found to represent 8±2% of total parasite GAPDH activity.

### GAPDH is found in the schistosome tegument

**Fig. 2.**
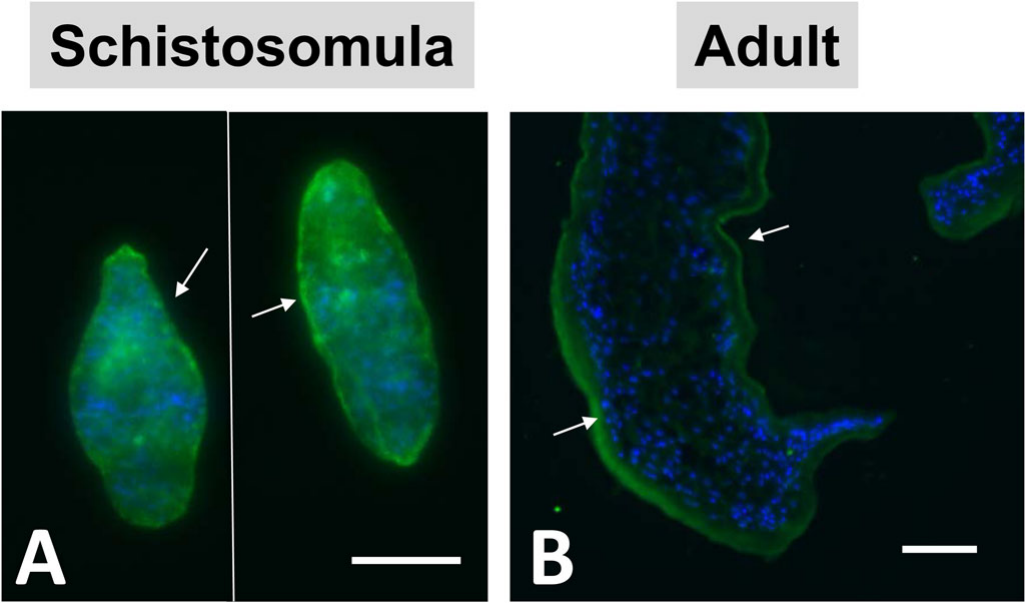
**Immunolocalization of SmGAPDH in *S. mansoni* schistosomula and adult worms.** (A,B) Indirect immunofluorescent labeling of native SmGAPDH (green) in whole fixed schistosomula (A) and sections of adult *S. mansoni* worms (B) using anti-GAPDH IgG as the primary antibody and rabbit anti-mouse IgG fragments conjugated to Alexa-488 as the secondary antibody. DAPI counterstaining was applied to highlight nuclei (blue). Arrows indicate the tegumental localization of GAPDH. Scale bars: 50 μm. Immunolocalization experiments were replicated at least two times.

To immunolocalize GAPDH, whole, fixed schistosomula (cultured for 7 days) and sections of adult male parasites were stained with mouse anti-GAPDH IgG antibody. In both life stages, some staining was seen throughout the body, but stronger staining was evident in the tegument of schistosomula (Fig. 2A, arrows) as well as in the adult parasite tegument (Fig. 2B, arrows). Control parasites, incubated with secondary antibody alone, did not exhibit staining (not shown).

### Suppression of SmGAPDH gene expression using RNAi interference (RNAi)

**Fig. 3.**
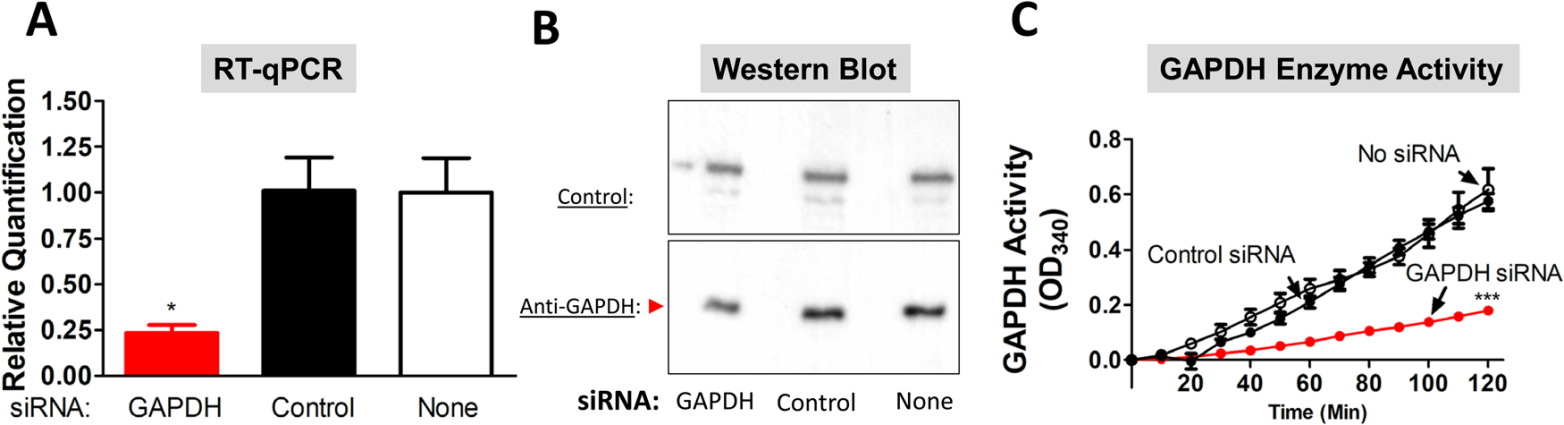
**SmGAPDH gene suppression using RNA interference.** (A) Relative SmGAPDH gene expression (mean±s.e.m., *n*=3) in schistosomula 72 h after electroporation with siRNA targeting SmGAPDH (red bar), control siRNA (black bar) or no siRNA (white bar, None) as assessed by RT-qPCR. Significant differences are denoted by *P<0.05 (one-way ANOVA with Tukey's post-test). (B) Western blot analysis of SmGAPDH protein levels in schistosomula homogenates 7 days after parasite treatment with siRNA targeting SmGAPDH (left) or control siRNA (middle) or no siRNA (right, None) as indicated. The SmGAPDH band is indicated in the lower panel (red arrowhead). The blot was additionally probed with an in-house, control antibody (upper panel) targeting *S. mansoni* acetylcholinesterase to show that all lanes received roughly equivalent amounts of parasite protein. (C) Mean surface SmGAPDH activity (NADH generation measured as mean of triplicate OD_340_ values±s.e.m.) in live schistosomula (in groups of 1000) tested 7 days after treatment with siRNA targeting SmGAPDH (red line), control siRNA or no siRNA, as indicated. Significant differences after 70 min are denoted by ***P<0.001 (two-way ANOVA with Bonferroni post-test). All knockdown-related experiments were replicated at least three times.

Fig. 3A shows that treatment of schistosomula with a small interfering RNA (siRNA) targeting the SmGAPDH gene leads to significant (∼80%) gene suppression, as measured by quantitative reverse transcription polymerase chain reaction (RT-qPCR), when compared with schistosomula treated with a control siRNA (Fig. 3A, Control) or schistosomula treated with no siRNA (Fig. 3A, None) (*P*<0.05 treated versus either control).

In addition, as shown by western blot analysis (Fig. 3B), appreciably less SmGAPDH protein can be detected in extracts of parasites treated with an siRNA targeting the GAPDH gene (red arrowhead) compared to parasites treated with a control siRNA or no siRNA, as indicated. The same worm extracts were probed with an in-house control antibody (Fig. 3B, Control, targeting *S. mansoni* acetylcholinesterase) to demonstrate that each extract contained roughly the same amount of protein. Finally, as shown in Fig. 3C, live parasites treated with the SmGAPDH siRNA display significantly less surface GAPDH enzyme activity (red line) compared to parasites treated with control siRNA or no siRNA (black lines) (*P*<0.001 for all time points beyond 70 min).

Despite robust GAPDH gene suppression, parasites treated with the SmGAPDH siRNA displayed no appreciable difference in morphology, motility or viability throughout the course of these experiments.

### Heterologous expression, purification and characterization of recombinant SmGAPDH (rSmGAPDH)

**Fig. 4.**
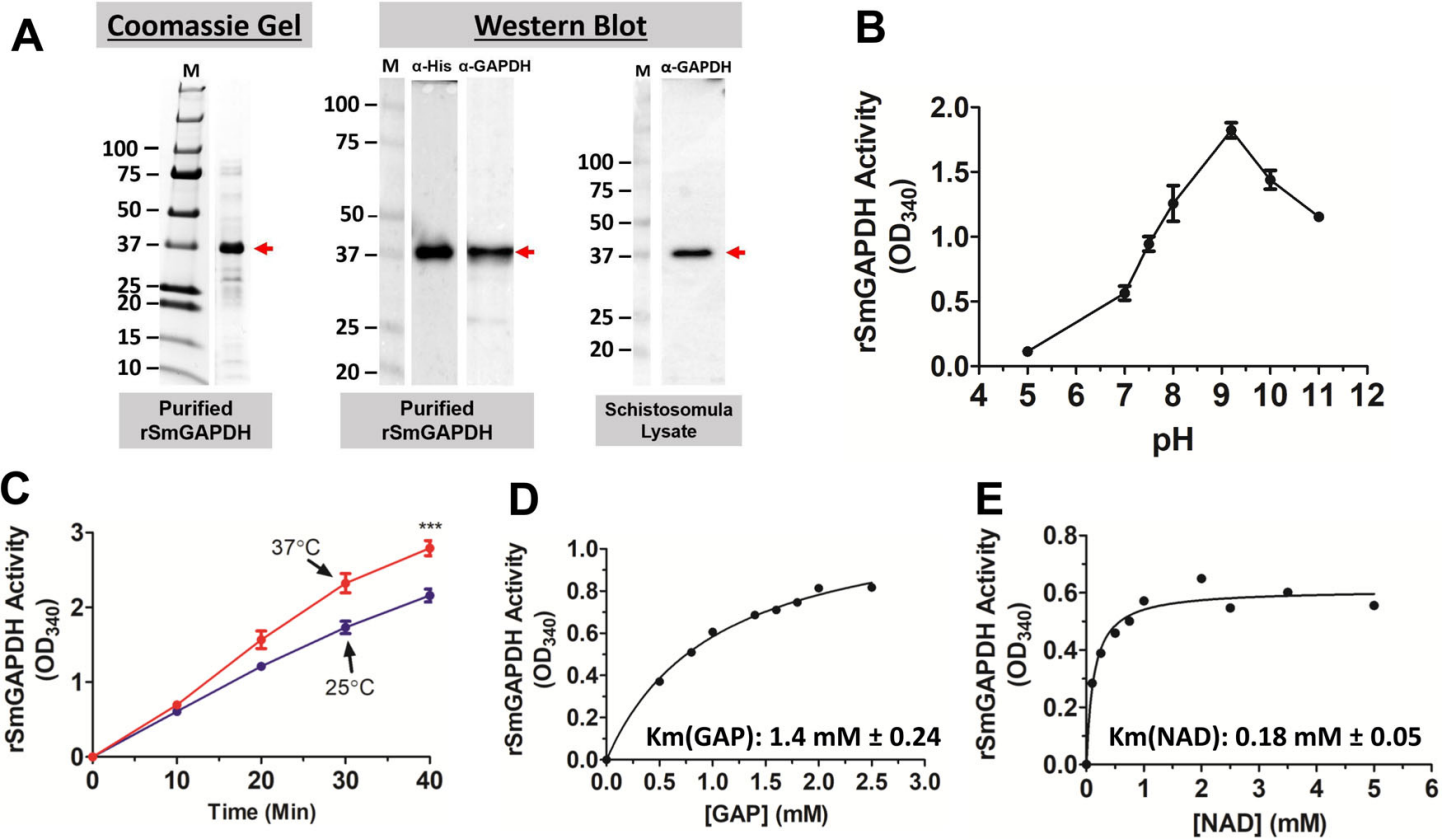
**Heterologous expression, purification and characterization of rSmGAPDH.** (A) Coomassie Blue-stained gel (left) showing purified rSmGAPDH running at ∼37 kDa (red arrow). Western blot analysis (center) indicating pure rSmGAPDH as detected using anti-His (α-His) and anti-GAPDH (α-GAPDH) antibodies (red arrow). Schistosomula lysate (right) was resolved by SDS-PAGE, blotted to PVDF membrane and probed with anti-GAPDH antibody. A single prominent protein running at the expected size of SmGAPDH is detected (red arrow). ‘M’ indicates molecular markers and numbers represent kDa. (B) Recombinant SmGAPDH activity (mean of triplicate OD_340_ values±s.e.m. at 120 min) in buffers for which pH ranges from 5 to 11. Maximal activity is observed at pH 9.2. (C) Recombinant SmGAPDH activity (mean of triplicate OD_340_ values±s.e.m. measured over time) at 25°C and 37°C, representing snail and mammalian host temperatures, respectively. ***P<0.001 (two-way ANOVA with Bonferroni post-test). (D,E) Michaelis–Menten kinetic curves are shown for the two co-substrates of rSmGAPDH: GAP (D) and NAD (E). The K_m_ values shown are the means from four independent experiments (activity is based on NADH generation, mean OD_340_±s.e.m. at 10 min). All rSmGAPDH characterization experiments were replicated at least three times.

A plasmid containing the SmGAPDH coding DNA (GenBank accession number XM_018794048.1), and an in-frame poly-his motif, was expressed in DS113 *Escherichia* *coli* (Coli Genetic Stock Center, New Haven, CT, USA). In this strain, the *gapA* and *gapB* genes have been deleted, and the bacteria lack detectable GAPDH activity (Seta et al., 1997). rSmGAPDH protein was purified by standard immobilized metal affinity chromatography (IMAC). Fig. 4A (left) shows an aliquot of the purified protein resolved by sodium dodecyl sulfate–polyacrylamide gel electrophoresis (SDS-PAGE) and stained with Coomassie Blue. A prominent band is seen at ∼37 kDa (Fig. 4A, left, arrow) and this is the expected size of rSmGAPDH. This protein is also detected by western blot analysis of the purified protein when probed with either an anti-his-tag antibody (α-His) or an anti-GAPDH (α-GAPDH) antibody (Fig. 4A, center, arrow). Fig. 4A (right) shows an *S. mansoni* schistosomula protein lysate resolved by SDS-PAGE and subjected to western blot analysis using the anti-GAPDH antibody. A protein of the expected size of GAPDH (37 kDa) is detected (Fig. 4A, right, arrow).

Fig. S1 shows the results from attempts to purify rSmGAPDH following expression in the *E. coli* strain BL21 Star (DE3)*.* Two prominent proteins are seen – one running at ∼37 kDa (Fig. S1, left panel, red arrowhead) and the other at ∼35 kDa (Fig. S1, left panel, black arrow). Both proteins are detected using anti-GAPDH antibody (Fig. S1, right panel, α-GAPDH, black arrow), whereas only the 37 kDa band is detected using an anti-his-tag antibody (Fig. S1, right panel, α-His, red arrowhead). The molecular weight of *E. coli* is reported to be 35 kDa (D'Alessio and Josse, 1971). We interpret our data to mean that only the upper 37 kDa band is rSmGAPDH while the lower 35 kDa band is *E. coli* GAPDH, which has dimerized with rSmGAPDH and is thus co-purified (Butterfield et al., 2010).

Fig. 4B–E show the results of our biochemical characterization of rSmGAPDH. The purified protein exhibits optimal activity at pH 9.2 (Fig. 4B). Although it shows robust activity at both 25°C and 37°C, higher activity is seen at 37°C (Fig. 4C, *P*<0.05 at all time points beyond 20 min). Adding calcium or magnesium (to 5 mM) to the reaction mixture, as well as removing divalent ions by adding the chelating agent EDTA to the reaction mixture, did not significantly affect enzyme activity (not shown).

To assess SmGAPDH enzyme kinetics, Michaelis–Menten curves were generated, yielding a Michaelis–Menten constant (K_m_) for GAP of 1.40±0.24 mM (Fig. 4D), and for NAD of 0.18±0.05 mM (Fig. 4E).

### rSmGAPDH binds to and enhances activation of plasminogen

**Fig. 5.**
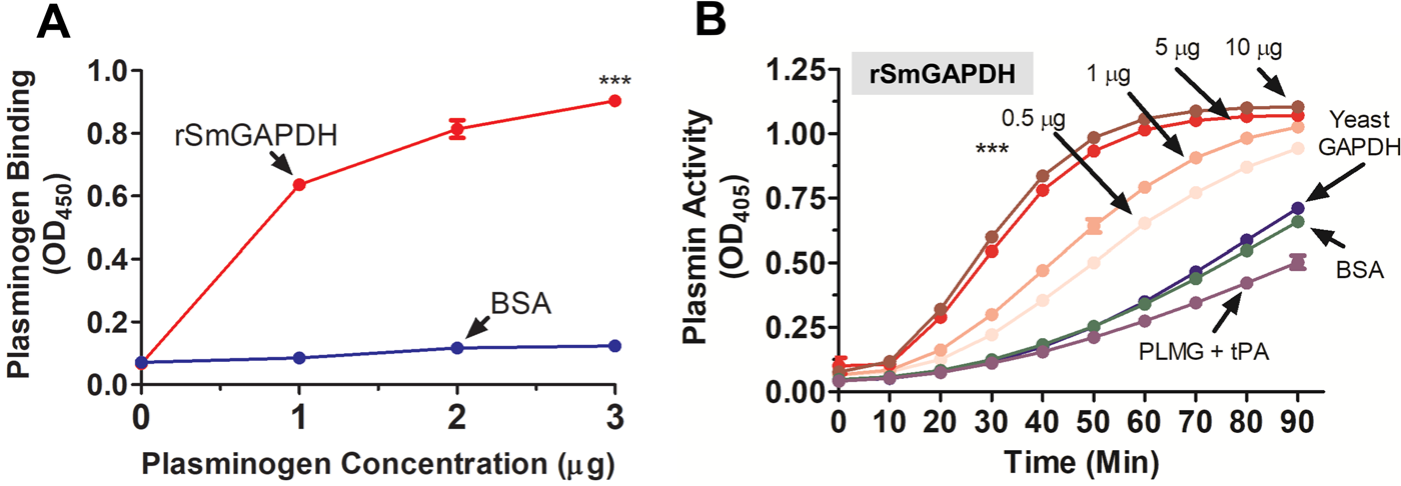
**Recombinant SmGAPDH binds to plasminogen and enhances plasmin generation.** (A) Plasminogen binding to rSmGAPDH (0.5 μg, red line) versus control protein BSA (0.5 μg, dark blue line) detected by ELISA (mean of triplicate OD_450_ values±s.e.m.). Significant difference between plasminogen binding to rSmGAPDH and BSA at all assay points are denoted by ***P<0.001. Plasminogen binding experiments were replicated two times. Blue dots are symbols for BSA. (B) Plasmin activity (mean of triplicate OD_405_ values±s.e.m.) detected in the presence of tPA and plasminogen (PLMG) alone (purple line) or supplemented with increasing amounts of rSmGAPDH (0.5–10 μg, as indicated by light pink, coral, red and brown lines), or yeast GAPDH (5 μg, dark blue line) or control protein BSA (5 μg, green line) over time. Significant differences at all time points beyond 30 min are denoted by ***P<0.001 (two-way ANOVA with Bonferroni post-test). Plasmin activation assays were replicated at least three times.

The ability of rSmGAPDH to bind to plasminogen was tested by enzyme-linked immunosorbent assay (ELISA). Fig. 5A shows increased rSmGAPDH binding as the concentration of plasminogen is increased. Control protein (bovine serum albumin, BSA) exhibits no such binding (*P*<0.001 at all PLMG concentrations).

Fig. 5B shows that adding tPA to plasminogen leads, as expected, to plasmin formation (Fig. 5B, purple line, PLMG+tPA). When rSmGAPDH is added to this mixture, plasmin generation is significantly enhanced in a dose-response manner (Fig. 5B, light pink, coral, red and brown lines, *P*<0.001 at all concentrations and all time points beyond 30 min). Control protein (BSA, green line) and commercially obtained *Saccharomyces cerevisiae* (yeast) GAPDH (dark blue line), in the presence of tPA, minimally impact plasmin formation in this assay.

## DISCUSSION

While glycolysis is a conserved biochemical pathway that takes place in the cytosol of cells, several enzymes that drive this pathway have been reported to be present at the schistosome surface following proteomic analysis (reviewed by Pirovich et al., 2019). The glycolytic enzymes enolase and GAPDH were both identified as schistosome surface proteins in ten of 11 independent schistosome tegumental proteomic studies (Pirovich et al., 2019). No other glycolytic enzymes were found more frequently in the schistosome tegumentome. Earlier, we characterized enolase from *S.* *mansoni* (Figueiredo et al., 2015) and here we focus on *S. mansoni* GAPDH.

Using a heterologous anti-GAPDH antibody for immunolocalization, we confirm that the protein is found widely within the tissues of schistosomula and adult worms. More pronounced staining with anti-GAPDH antibody was observed in the parasite tegument. This pattern of distribution is not a surprise since the tegument, like all tissues, is expected to engage in glycolysis, which requires GAPDH. More striking is our finding that intact, living schistosomes express functional GAPDH on their external surface: adding GAP plus NAD in the presence of live worms yields NADH, which is detected spectrophotometrically. This result is consistent with the conversion of GAP and NAD to 1,3-bisphosphoglycerate and NADH by active GAPDH on the worms. All intravascular life stages tested (schistosomula and adult males and females) express this trait. Since some worms are known to secrete GAPDH, e.g. the ruminant nematode *Haemonchus contortus* (Rajan et al., 2018; Sahoo et al., 2013; Vedamurthy et al., 2015), and GAPDH has been detected in the excretory-secretory products of some schistosome life stages (Knudsen et al., 2005; Liu et al., 2009; Mathieson and Wilson, 2010), we set out to assess whether the *S. mansoni* GAPDH enzyme activity detected might be due primarily to protein being secreted by the worms (or protein that was released from worms because of damage in culture). To do this, we compared GAPDH activity in medium in which parasites had been cultured overnight to the activity displayed by the cultured worms themselves. We discovered that while the live worms (schistosomula or adult males or females) displayed robust GAPDH activity, the overnight culture medium showed just trace activity. This means that the enzyme is associated with the parasites and is not released in any appreciable quantity during the time course of this experiment. GAPDH has been reported to be present in exosome-like vesicles (ELVs) that are secreted by schistosomes (Samoil et al., 2018), but whether they are on the surface of the ELVs and/or within them has not been established. Schistosome GAPDH has been reported to be associated with the vascular endothelium of mice infected with *S. bovis*, suggesting that the protein can be released by *S. bovis* worms *in vivo* (de la Torre-Escudero et al., 2012). Not surprisingly, since glycolysis is a major cytosolic process, the surface GAPDH associated with live *S. mansoni* schistosomula reported here represents a small fraction (∼8%) of that present in the entire worms.

Our results confirm an association of SmGAPDH with the tegumental surface of the worm. This further reconciles results that previously characterized SmGAPDH as a protective vaccine target (Argiro et al., 2000b; El Ridi and Tallima, 2013; Tallima et al., 2017; Tang et al., 2019). Before these results, how such an intracellular cytosolic enzyme might be accessed by damaging immune effectors was a conundrum. Our demonstration that SmGAPDH is both intracellular and exposed on the parasite surface, making it potentially accessible to immune cell and antibody interactions, provides a solution. The mechanisms, which allow SmGAPDH, and other proteins like it that lack conventional signal sequences, to be trafficked to the parasite surface membrane are still unknown and require investigation.

In this work, expression of the SmGAPDH gene was successfully suppressed using RNAi; significantly lower GAPDH mRNA levels were measured in schistosomula treated with siRNAs targeting GAPDH versus controls, as measured by RT-qPCR. Suppressed worms had notably less GAPDH protein as detected by western blot analysis, and they displayed significantly lower surface GAPDH enzyme activity compared to controls. Despite this, and despite the presumed importance of an active glycolytic pathway for the worms, SmGAPDH-suppressed worms displayed no overt change in morphology or viability compared to control worms. The same phenomenon was observed following suppression of the *S. mansoni* enolase gene in schistosomula. We conclude that the worms do not require a fully functional glycolytic pathway to remain viable, at least in culture. In contrast, GAPDH knockdown in cancer cells leads to cell death (Phadke et al., 2009).

In order to begin to assess possible functions for surface-associated schistosome GAPDH, this protein was first generated in recombinant form following expression in *E. coli*. Initial attempts utilized strain BL21 Star (DE3), which yielded two prominent purified protein products – SmGAPDH and *E.* coli GAPDH. Since we were eager to examine just SmGAPDH, we elected to express the protein in an *E.* *coli* strain in which GAPDH genes were deleted (strain DS113), and this approach yielded a single purified protein of the expected size of SmGAPDH. The tendency of conserved GAPDH monomers to multimerize (Butterfield et al., 2010) suggests that previous attempts to express and purify heterologous GAPDH enzymes in *E.* *coli* strains other than DS113 may have yielded impure preparations and could call into question the validity of any subsequent enzyme characterization.

Recombinant SmGAPDH purified from *E. coli* strain DS113 was enzymatically active and showed robust activity at a range of temperatures, including the body temperature of both the mammalian definitive host (∼37°C) and the snail intermediate host (∼25°C). In agreement with a previous report (Argiro et al., 2000a), SmGAPDH displays optimal enzyme activity at a pH of ∼9.2, suggesting that, in the relatively neutral pH of the bloodstream, any surface SmGAPDH activity might be tempered. It is noteworthy that several surface-expressed schistosome enzymes also display alkaline pH optima (Cesari et al., 1981; Da'dara et al., 2014; Elzoheiry et al., 2018a). SmGAPDH, like GAPDH enzymes from other species, displays no requirement for divalent ions for its catalytic activity (Maurer et al., 2015; Kim et al., 2013). The K_m_ measurements derived for the enzyme for its substrates GAP (∼1.4 mM) and NAD (∼0.2 mM) are both within the ranges reported for GAPDH enzymes of other organisms (Sangolgi et al., 2016; Zinsser et al., 2014).

What might be the function of extracellular, tegument-associated SmGAPDH? Is there any selective advantage for the parasites to express the protein in this location? In schistosomes, and in other systems, evidence is accumulating that extracellular glycolytic enzymes like GAPDH can be engaged in non-traditional, non-glycolytic or ‘moonlighting’ functions relating, for example, to immune modulation and/or blood clot dissolution (Karkowska-Kuleta and Kozik, 2014; Pirovich et al., 2019; Sirover, 2017). We previously showed that live intravascular schistosomes could promote PLMG activation in the presence of tPA (Figueiredo et al., 2015). Further, SmEno, a glycolytic enzyme found widespread within schistosome internal tissues but also found at the host parasite interface likewise can (in recombinant form) activate PLMG to generate plasmin (Figueiredo et al., 2015). Here, we show that SmGAPDH is a second glycolytic enzyme found at the *S. mansoni* surface that can similarly bind plasminogen and that can promote its conversion to plasmin in a dose-dependent manner. Neither the control protein BSA nor GAPDH from the yeast *Saccharomyces cerevisiae* exert this effect. GAPDH and enolase are among the surface-associated antigens of the intravascular nematode parasite *Dirofilaria immitis* that bind PLMG (González-Miguel et al., 2013); recombinant *D. immitis* GAPDH has also been shown to stimulate plasmin generation by tPA (González-Miguel et al., 2015). Recombinant *Onchocerca volvulus* GAPDH binds PLMG (Erttmann et al., 2005), as does surface-localized GAPDH from the Chinese liver fluke *Clonorchis sinensis* (Hu et al., 2014). In *S.* *bovis*, several glycolytic enzymes (including GAPDH and enolase) were identified in tegumental extracts that could bind PLMG (Ramajo-Hernández et al., 2007). However, unlike the situation reported here for rSmGAPDH, recombinant *S. bovis* GAPDH, while binding to PLMG, did not potentiate its conversion to plasmin in the presence of tPA (de la Torre-Escudero et al., 2017). This is surprising, given the high degree of conservation between SmGAPDH and its homolog in *S. bovis* (92% identity), and suggests that subtle conformational differences between various GAPDHs are responsible for the different capabilities noted.

By recruiting host PLMG and promoting its conversion to plasmin, surface SmGAPDH could help drive the degradation of any blood clots forming around schistosomes in the vasculature thus allowing them unrestricted movement *in vivo*. This finding adds to our knowledge concerning the ability of schistosomes to control hemostasis: the worms have previously been reported to possess a series of ectoenzymes that likely impact blood clot formation by degrading host prothrombotic signaling molecules as well as key coagulation proteins (Elzoheiry et al., 2018b; Leontovyč et al., 2018; Mebius et al., 2013; Wang et al., 2018, 2017).

Extracellular GAPDH enzymes from other systems can engage in functions unrelated to PLMG binding and thrombus formation. For example, GAPDH is a major surface protein in streptococci bacteria that can act in a variety of different ways: it can be an ADP-ribosylating enzyme (Pancholi and Fischetti, 1993), it can induce the proliferation and differentiation of B cells *in vitro* (Madureira et al., 2007), it can bind multiple host proteins (fibronectin, actin, lysosome) (Jin et al., 2011), and, when injected intraperitoneally into mice, it can quickly cause serum IL-10 levels to rise (Madureira et al., 2007). Additional novel functions ascribed to recombinant streptococcus GAPDH include its ability to inhibit both complement component C5a-activated chemotaxis and hydrogen peroxide production in human neutrophils (Terao et al., 2006) as well as its ability to induce apoptosis in murine macrophages (Oliveira et al., 2012). Finally, a *Streptococcus agalactiae* strain overexpressing GAPDH exhibited increased virulence in mice when compared with wild-type bacteria (Oliveira et al., 2012). Thus, streptococcal GAPDH is increasingly recognized as an important virulence factor and this raises the possibility that schistosome surface-bound GAPDH might exert similar diverse functions to promote the growth and survival of these debilitating blood parasites.

There is also evidence that pathogenic organisms use their surface-bound GAPDH as a transferrin (Tf)-binding protein, facilitating the transport of extracellular iron – an essential element in several biological functions (Boradia et al., 2014; Modun and Williams, 1999). Schistosomes require iron for their growth, development and reproduction (Glanfield et al., 2007); it was hypothesized that schistosomula growth is stimulated by a non-specific surface binding of Tf-bound iron (Clemens and Basch, 1989). There are currently no described Tf receptors on the schistosome tegument (Glanfield et al., 2007), which leads us to hypothesize that tegumental GAPDH might be serving an additional moonlighting function in facilitating iron uptake.

## MATERIALS AND METHODS

### Parasites and mice

Cercariae of *S. mansoni* (NMRI) shed from infected *Biomphalaria glabrata* snails (strain NMRI, NR-21962, Schistosomiasis Resource Center, at the Biomedical Research Institute in Rockville, MD, USA), were manually transformed into schistosomula and cultured for at least 1 week *in vitro* as previously described (Da'dara and Skelly, 2015). Adult worms were obtained following perfusion of female 6- to 8-week-old Swiss-Webster mice (*Mus musculus*) 6–7 weeks post-infection. All parasites were cultured in complete Dulbecco's modified Eagle's medium (DMEM)/F12 medium [supplemented with 10% heat-inactivated fetal bovine serum, 200 U/ml penicillin and 200 μg/ml streptomycin, 0.2 μM Triiodo-L-thyronine (T_3_), 1 μM serotonin and 8 μg/ml human insulin] and were maintained at 37°C in an atmosphere of 5% CO_2_ (Milligan and Jolly, 2011). All protocols involving animals were approved by the Tufts University Institutional Animal Care and Use Committee (IACUC) under protocol G2018-68. We confirm that the care and use of experimental animals complied with all relevant local animal welfare laws, guidelines and policies.

### Characterization of native *S. mansoni* GAPDH

Live, intact parasites (500 schistosomula or two adult males or females per well, in replicate) were washed and resuspended in 100 μl assay buffer (100 mM NaPO_4_, 80 mM triethanolamine, 0.2 mM EDTA, pH 9.2). The enzyme reaction was started by the addition of 100 μl substrate (10 mM NAD, 10 mM GAP) in assay buffer. GAPDH activity was monitored by the generation of NADH using a Synergy HT microplate spectrophotometer (BioTek, Winooski, VT, USA) at OD_340_ over time. Negative controls included adding individual components of the substrate mixture to the parasites. In some experiments, 500 schistosomula parasites were manually homogenized in Hank's Balanced Salt Solution (HBSS) and GAPDH activity was measured in aliquots of this lysate as described above.

To investigate the possibility that GAPDH was secreted or released by the parasites *in vitro*, 500 schistosomula or male and female adult worms in replicate were washed in HBSS and incubated overnight in 500 μl clear DMEM (supplemented with 200 U/ml penicillin and 200 μg/ml streptomycin). After overnight incubation, the conditioned medium was separated from the worms and an equal volume of assay buffer (100 mM NaPO_4_, 80 mM triethanolamine, 0.2 mM EDTA, pH 9.2) containing 10 mM NAD, 10 mM GAP, was added. GAPDH activity in this overnight conditioned medium was monitored by measuring NADH generation, as above. The overnight incubated worms were resuspended in fresh clear DMEM to which an equal volume of assay buffer containing 10 mM NAD, 10 mM GAP, was added and any GAPDH activity was measured, as just described.

### SmGAPDH gene suppression via RNAi

The following two SmGAPDH-specific siRNAs were commercially synthesized (Integrated DNA Technologies) and used at 6 μM for SmGAPDH gene expression knockdown: SmGAPDH siRNA #1, 5′-GGTCATTCATGATAAGTTTGAAATA-3′; SmGAPDH siRNA #2, 5′-CGAGCTAAAAAGGTCATAATATCTG-3′. These siRNAs or a control siRNA, described previously (Da'dara and Skelly, 2015), were delivered to schistosomula (∼2000 per treatment) or adult parasites (eight to ten worms per treatment) by electroporation as previously reported (Da'dara and Skelly, 2015).

RT-qPCR was performed using TaqMan Assays 72 h post-siRNA administration. The following primer sets and reporter probe customized for SmGAPDH were purchased from Life Technologies (Carlsbad, CA, USA): F-primer, 5′-AGTCTACTGGAGTCTTTACGACCAT-3′; R-primer, 5′-TGCAGATATTATGACCTTTTTAGCTCGAT-3′; FAM-probe, 5′-ATGAGCCTGAGCTTTATC-3′.

As an endogenous control, we used the housekeeping tubulin gene to compare relative SmGAPDH expression in live parasites, as previously reported (Figueiredo et al., 2015). Each RT-qPCR reaction was performed using 2 μl of complementary DNA (cDNA) in a final volume of 20 μl. All reactions were run in triplicate in a StepOne Plus system (Life Technologies, Carlsbad, CA, USA). To determine relative quantification, the ΔΔCT method was utilized.

Seven days after siRNA treatment, live schistosomula (in groups of 1000 and in replicate) were monitored for their GAPDH activity following the assay described above. In addition, western blot analysis was conducted to measure changes in GAPDH protein levels. To do this, parasite extracts were first resolved on a 4–20% Mini-PROTEAN^®^ TGX™ SDS-polyacrylamide gel (Bio-Rad, Hercules, CA, USA). The proteins were then transferred to activated polyvinylidene fluoride (PVDF) membrane and blocked with PBST [phosphate buffered saline (PBS), pH 7.6, 0.05% Tween-20] containing 5% dry non-fat milk powder. To detect the presence of SmGAPDH, the membrane was incubated with a mouse monoclonal IgG1 GA1R anti-recombinant GAPDH antibody (1:2000, MA5-15738, Thermo Fisher Scientific, Rockford, IL, USA). Following a 1-h incubation at room temperature, the membrane was washed and incubated with goat anti-mouse IgG conjugated to horseradish peroxidase (HRP) (1:5000, STAR207P, Bio-Rad) for 1 h at room temperature. Blots were developed using ECL Western Blotting Detection Reagents (GE Healthcare Bio-Sciences, Piscataway, NJ, USA) according to the manufacturer's instructions. Western blot images were captured on a ChemiDoc Touch Imaging System (Bio-Rad). The membrane was stripped with Restore™ Western Blot Stripping Buffer (Thermo Fisher Scientific) and re-probed with an in-house control *S. mansoni* antibody as described above.

### Immunolocalization of SmGAPDH

Whole schistosomula cultured for 7 days were fixed in ice-cold acetone for 5 min at room temperature. Frozen sections (6-μm thick) of adult worms in OCT compound were thawed at room temperature, and a hydrophobic marker was used to circle the sectioned parasites. Sections were hydrated for 10 min with PBS, then washed with PBST and blocked with blocking buffer [1% bovine serum albumin (BSA) in PBS] for 1 h. Next, samples were treated with mouse monoclonal IgG1 GA1R anti-recombinant GAPDH antibody (Thermo Fisher Scientific, Rockford, IL, USA) diluted 1:100 for 1 h. After washing with PBS, samples were incubated for 1 h with F(ab)_2_ fragments of rabbit anti-mouse IgG antibody conjugated to Alexa-488 (A-21204, Invitrogen, Carlsbad, CA, USA) diluted in blocking buffer at 1:100. Finally, samples were incubated with 0.3 mM 4′,6-diamidino-2-phenylindole (DAPI) in PBS and 1% BSA for 5 min, washed in PBST, mounted in fluoromount and viewed on an inverted fluorescent microscope (Eclipse Ti, Nikon, Tokyo, Japan).

### Cloning and expression of recombinant *S. mansoni* GAPDH (rSmGAPDH)

To generate a PCR fragment containing the entire SmGAPDH coding DNA based on the published GAPDH coding sequence (GenBank accession number XM_018794048.1), adult worm cDNA was used as a template and the following primers were utilized in the PCR: SmGAPDH-F, 5′-TTAACCGGATCCAATGTCGAGAGCAAAGGTTGGTATTAACGG-3′ and SmGAPDH-R, 5′-TCAAAACTCGAGTTATGCATGGTCGACTTTATGCATGTGCG-3′ containing BamHI and XhoI restriction sites, respectively (underlined). The PCR product was purified, digested with BamHI and XhoI and cloned into a pTrcHisB expression vector (Life Technologies) that had previously been digested with BamHI and XhoI. Successful generation of recombinant constructs was confirmed by DNA sequencing at GeneWiz (Cambridge, MA, USA). The resulting construct was transformed into BL21 Star (DE3) *E. coli* (Thermo Fisher Scientific) as well as GAPDH-deficient DS113 *E. coli* (Coli Genetic Stock Center, New Haven, CT, USA). Recombinant BL21 Star (DE3) transformants were cultured in Luria Broth supplemented with ampicillin (100 μg/ml). Recombinant DS113 transformants were cultured in Luria Broth supplemented with ampicillin (100 μg/ml), chloramphenicol (10 μg/ml) and erythromycin (25 μg/ml). Recombinant SmGAPDH (rSmGAPDH) protein production was induced using 1 mM isopropyl β-d-1-thiogalactopyranoside (IPTG) and the protein was purified using standard IMAC on a Ni-NTA Sepharose column according to the manufacturer's instructions (Life Technologies).

Protein fractions containing purified rSmGAPDH were dialyzed against PBS overnight at 4°C using Slide-A-Lyzer™ Dialysis Cassettes (Thermo Fisher Scientific) and concentrated using a 10 K MWCO Pierce™ Protein Concentrator (Thermo Fisher Scientific). Purified protein was quantified using a BCA kit (Pierce, Waltham, MA, USA). Protein fractions were resolved by 4-20% SDS-PAGE and stained with Coomassie Blue; gel images were captured on a ChemiDoc™ Touch Imaging System (Bio-Rad).

Purification of rSmGAPDH was confirmed by western blotting. Purified protein extracts were first resolved on a 4–20% Mini-PROTEAN^®^ TGX™ SDS-polyacrylamide gel (Bio-Rad). The proteins were then transferred to activated PVDF membrane and blocked with PBST containing 5% dry non-fat milk powder. To detect GAPDH, the membrane was incubated with a mouse monoclonal IgG1 GA1R anti-recombinant GAPDH antibody (1:2000, MA5-15738, Thermo Fisher Scientific). Following a 1-h incubation at room temperature, the membrane was washed and incubated with goat anti-mouse IgG conjugated to HRP (1:5000, STAR207P, Bio-Rad) for 1 h at room temperature. Blots were developed using ECL Western Blotting Detection Reagents (GE Healthcare Bio-Sciences) according to the manufacturer's instructions. Western blot images were captured on a ChemiDoc Touch Imaging System (Bio-Rad).

To detect the 6-histidine (His) protein tag, the membrane was incubated with a mouse monoclonal His-probe IgG antibody conjugated to HRP (Lot B1418, Santa Cruz Biotechnology, Dallas, TX, USA) at 1:2000. Blots were developed using ECL Western Blotting Detection Reagents (GE Healthcare Bio-Sciences) according to the manufacturer's instructions.

### rSmGAPDH enzyme characterization

Functional activity of rSmGAPDH was routinely monitored at 1 μg/well using the enzyme activity assay detailed above.

To determine the enzyme's pH optimum, activity was monitored under varying pH conditions (pH 5–11).

To test for temperature preferences, substrate and enzyme solutions were incubated at 25°C or at 37°C for 30 min before initiating the reaction by adding substrates (NAD and GAP at 5 mM final concentration) to the enzyme solution and measuring NADH generation at OD_340_ over time, as above. The impact of divalent ions on GAPDH activity was evaluated by varying their concentration in the reaction mixture (0–5.0 mM Mg^2+^ or Ca^2+^) to the reaction mixture, or by adding chelator, 10 mM EDTA.

To determine the K_m_ for the glycolytic substrate GAP, rSmGAPDH (1 μg/assay) activity was measured as described above and at a range of GAP substrate concentrations (0–10 mM) and 5 mM NAD. The K_m_ for NAD was calculated a similar manner, here using 0–10 mM NAD and 5 mM GAP. Data were analyzed and plotted using GraphPad Prism 5.0 (La Jolla, CA, USA).

### Plasminogen binding ELISA

To measure plasminogen binding to rSmGAPDH by ELISA, multi-well Nunc-Immuno microplates (Thermo Fisher Scientific) were first coated with 0.5 μg rSmGAPDH in carbonate-bicarbonate buffer (0.05 M, pH 9.6) and incubated overnight at 4°C. Wells coated with BSA in a similar manner served as controls. Next, blocking buffer (1% BSA in PBS supplemented with 0.05% Tween-20) was added to all wells for 1 h at 37°C. Zero to 3.0 μg plasminogen in blocking buffer was added to selected wells for 1 h at 37°C. All wells were then incubated with rabbit anti-plasminogen IgG antibody (PA5-34677, Invitrogen, Rockford, IL, USA) diluted 1:2000 in blocking buffer for 1 h at 37°C. HRP-conjugated donkey anti-rabbit IgG antibody (NA934V, GE Healthcare, Buckinghamshire, UK) diluted at 1:5000 in blocking buffer was added to all wells for 1 h at 37°C. Plates were developed by adding chromogenic substrate 3,3′,5,5′-Tetramethylbenzidine (TMB) (Thermo Fisher Scientific) to all samples for 2 min before stopping the reaction with 1 N HCl. Plates were read at OD_450_. Wells were washed with PBST between every step in the protocol.

### rSmGAPDH plasminogen activation assay

Recombinant SmGAPDH was tested for plasminogen activation as previously described (Figueiredo et al., 2015). Briefly, human plasminogen (3.0 μg, HYPHEN BioMed, Neuville-sur-Oise, France), recombinant human tPA (20 ng, HYPHEN BioMed, Neuville-sur-Oise, France) and rSmGAPDH or control protein [BSA and *Saccharomyces cerevisiae* (yeast) GAPDH] in PBS were added to microplate wells in a total volume of 150 μl. The synthetic plasmin substrate (4.5 μg D-Valyl-L-Leucyl-L-Lysine 4-nitroanilide dihydrochloride, Sigma-Aldrich, St Louis, MO, USA) was then added and any change in OD_405_ (indicative of substrate cleavage) was measured over time at 37°C using a spectrophotometer.

### Statistical analysis

Statistical analyses were performed using GraphPad Prism 5.0. For activity assays and plasminogen-activation assays, two-way analysis of variance (ANOVA) with Bonferroni post-tests were utilized. To compare the means of two samples, unpaired *t*-tests were used. To analyze RT-qPCR data, one-way ANOVA with Tukey's post-tests were employed. *P-*values were considered significant at <0.05.

## Supplementary Material

Supplementary information
