# Supplementary material for: Schistosoma mansoni glyceraldehyde-3-phosphate dehydrogenase enhances formation of the blood-clot lysis protein plasmin
Source: Biol Open. 2020 Mar 24;9(3):bio050385. doi: 10.1242/bio.050385 (PMC7104858; doi:10.1242/bio.050385)
Supplement: Supplementary information [file biolopen-9-050385-s1.pdf]

Biology Open (2020): doi:10.1242/bio.050385: Supplementary information

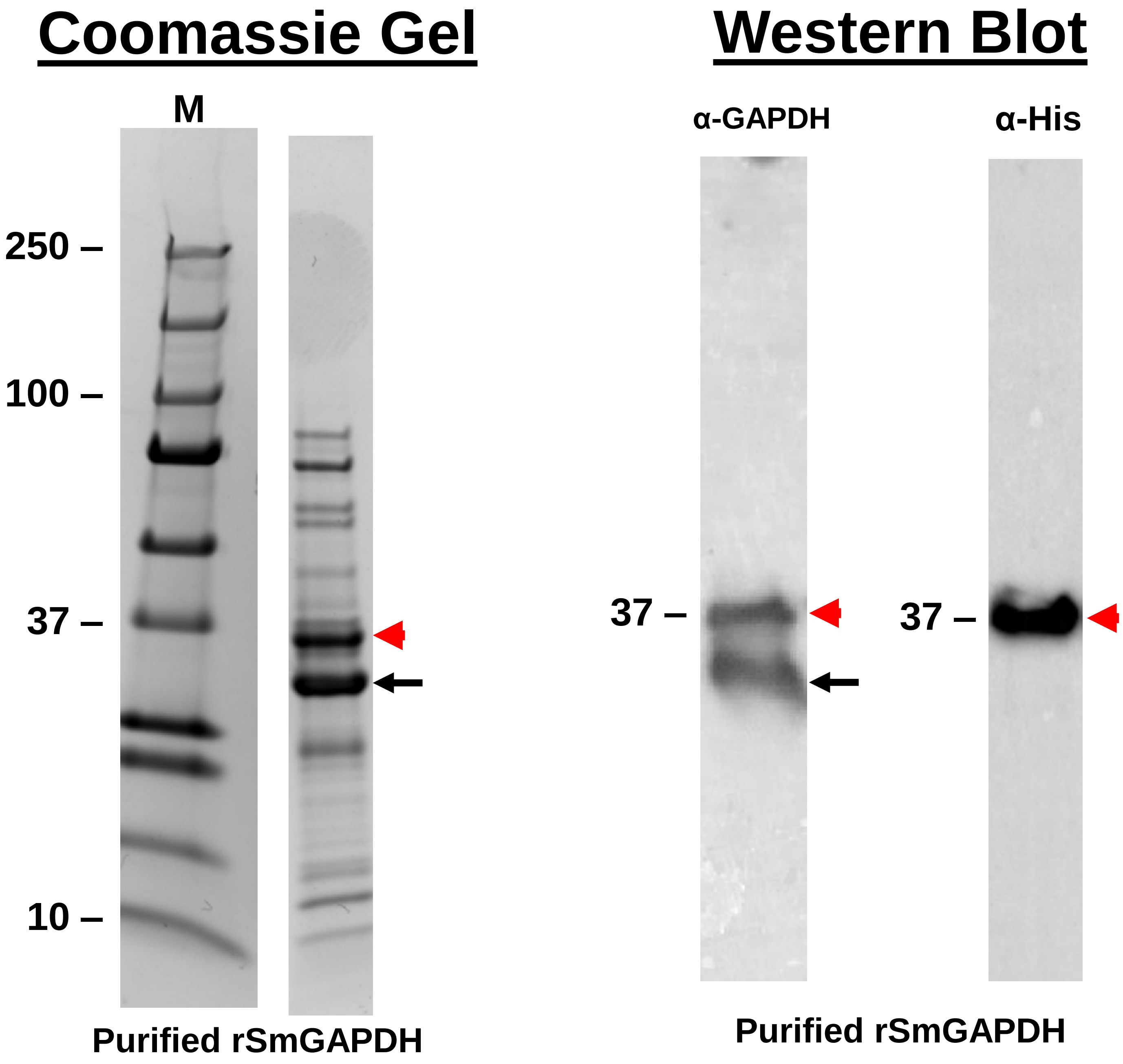

Figure S1. Expression of rSmGAPDH in BL21 Star (DE3) *E. coli*. Coomassie-stained gel (left panel) showing rSmGAPDH purified from transformed BL21 (DE3) *E. coli* running at ~37 kDa (red arrowhead) and *E. coli* GAPDH running at ~35 kDa (black arrow). Western blot analysis using anti-GAPDH antibody (α-GAPDH, right panel) detects both rSmGAPDH (~37 kDa, red arrowhead) and *E. coli* GAPDH (~35 kDa, black arrow). When probed with anti-his-tag antibody (α-His, right panel), a single band representing the his-tagged rSmGAPDH (~37 kDa, red arrowhead) is detected. "M" indicates molecular markers, and numbers represent kilodaltons (kDa). The molecular marker is identical to that in Figure 4A since all samples were run on the same gel.

Biology Open • Supplementary information
